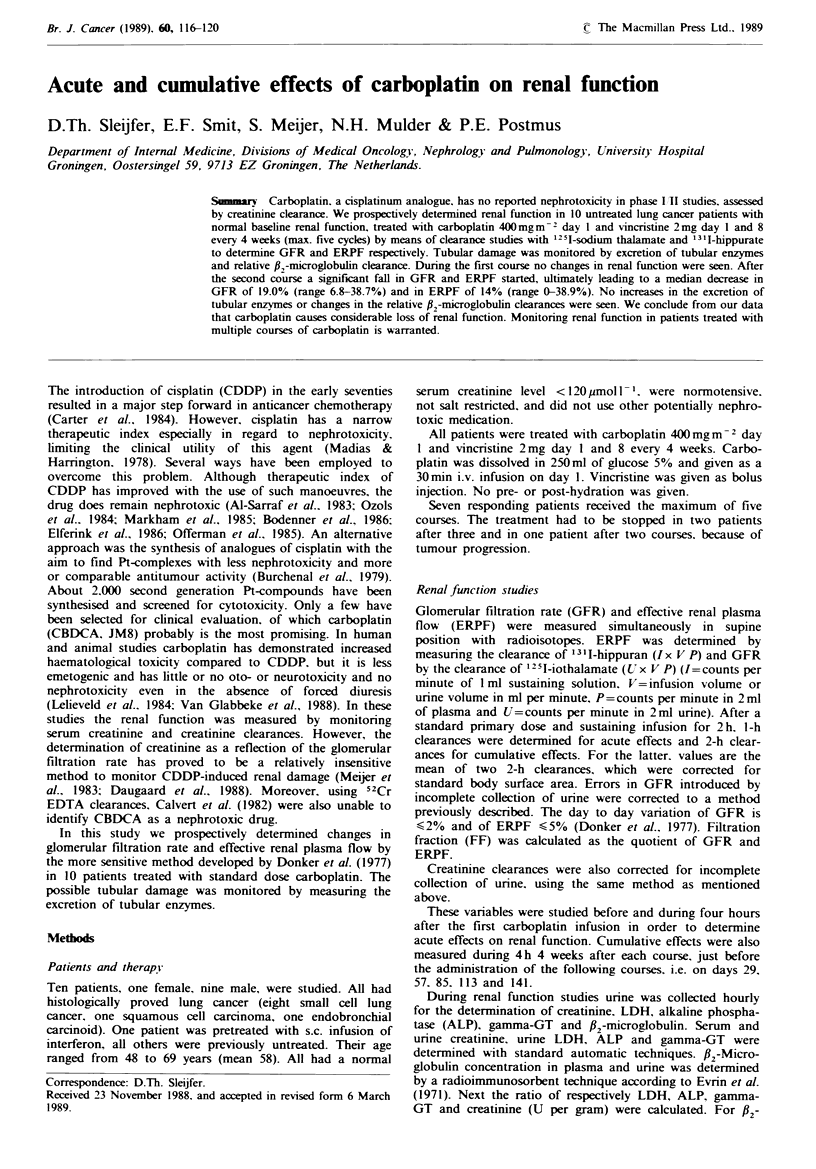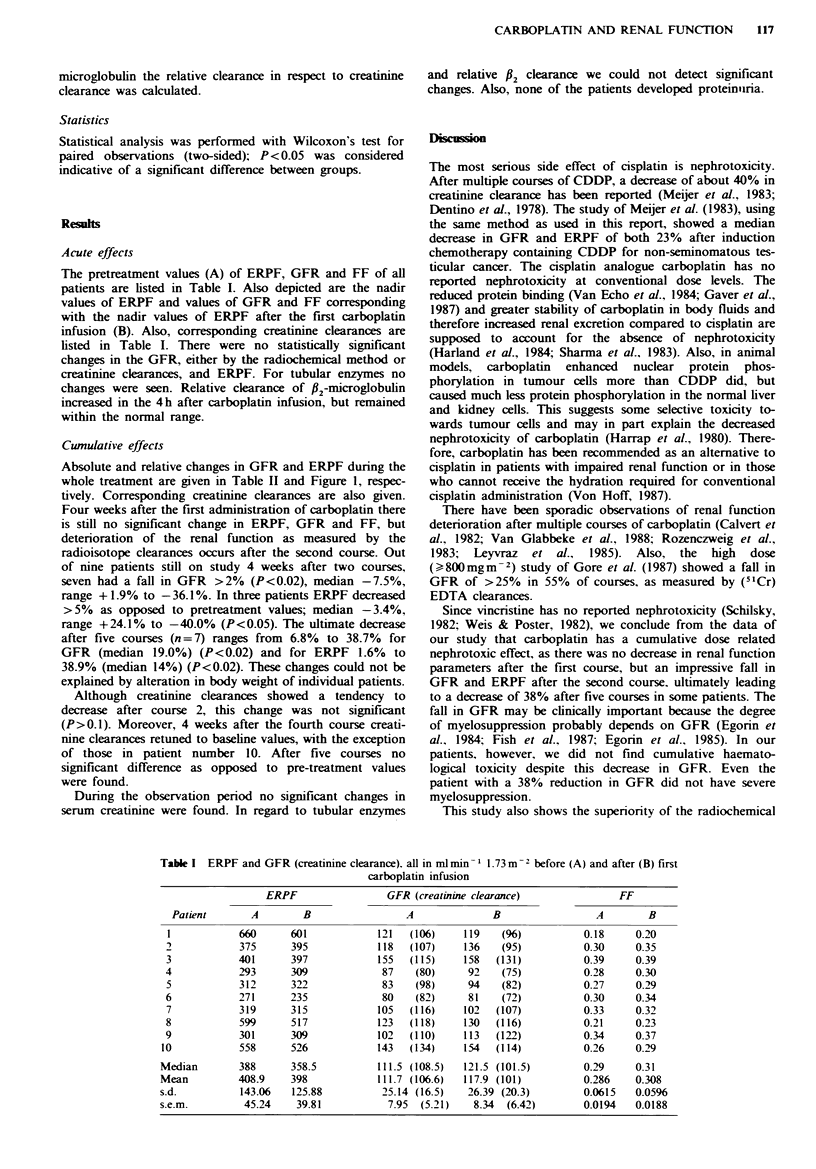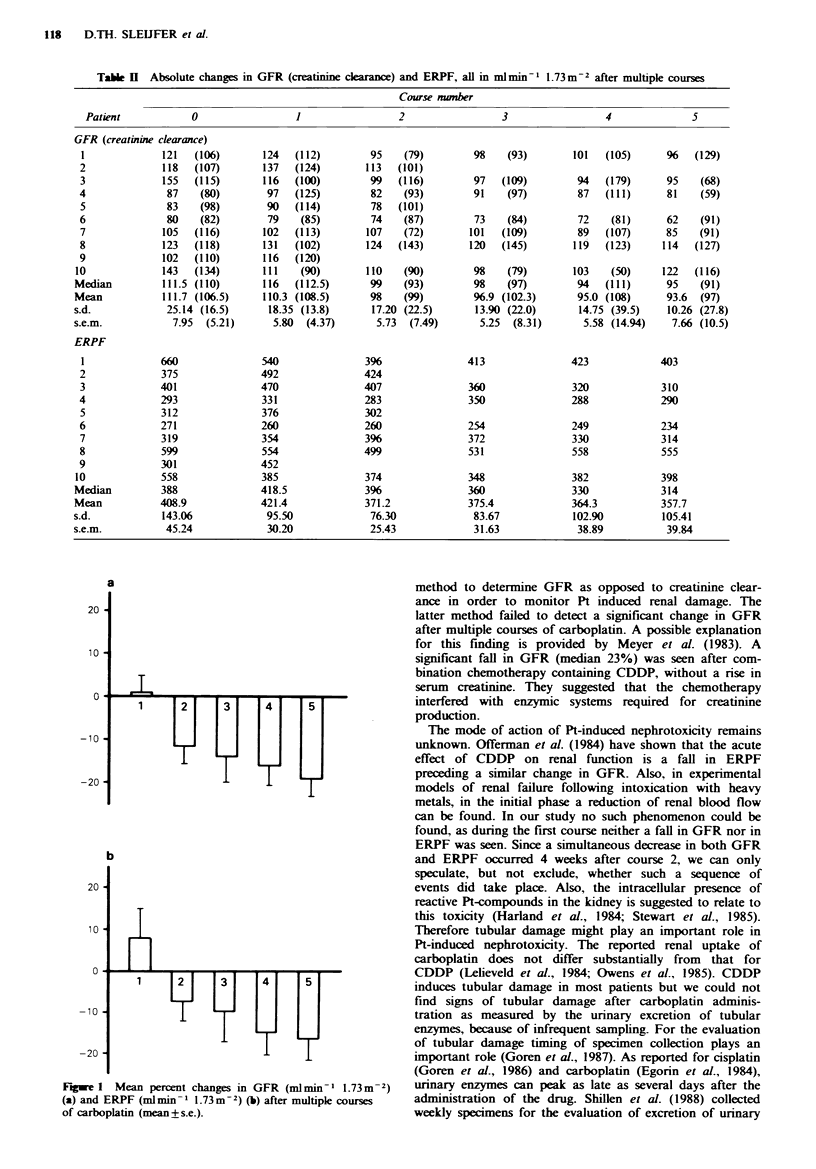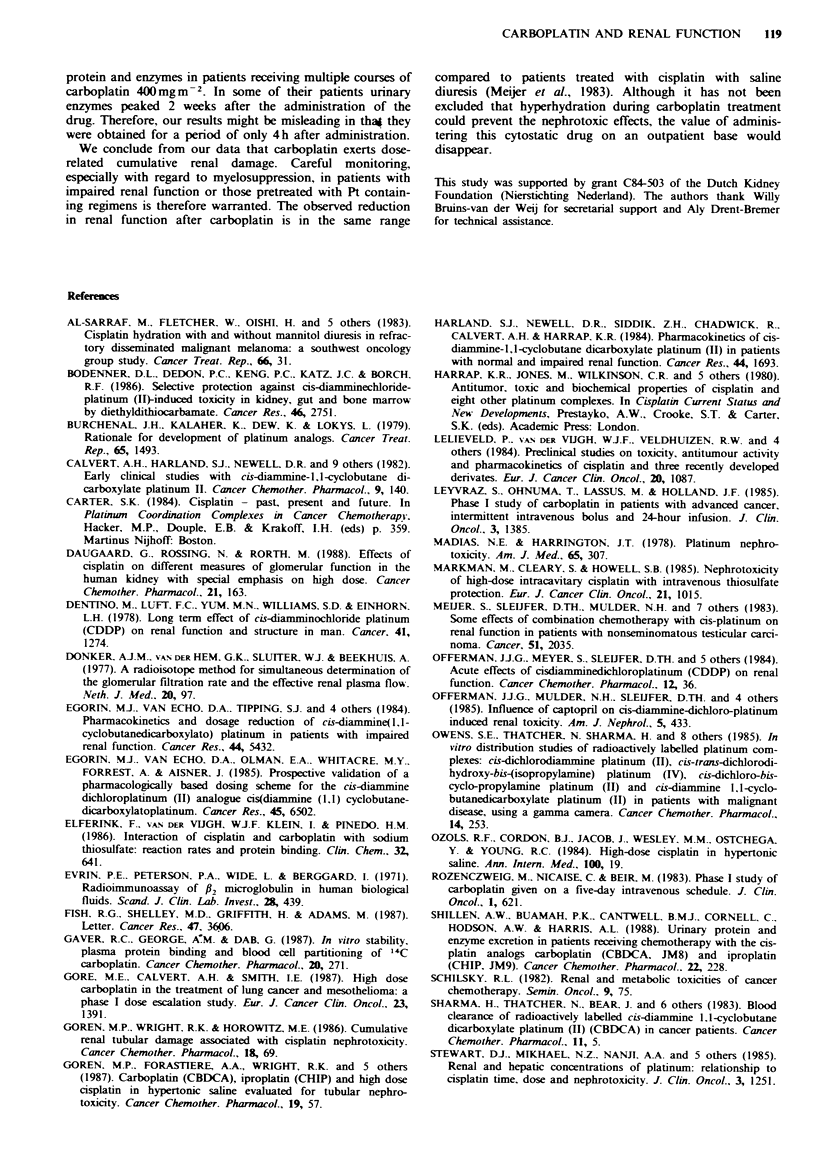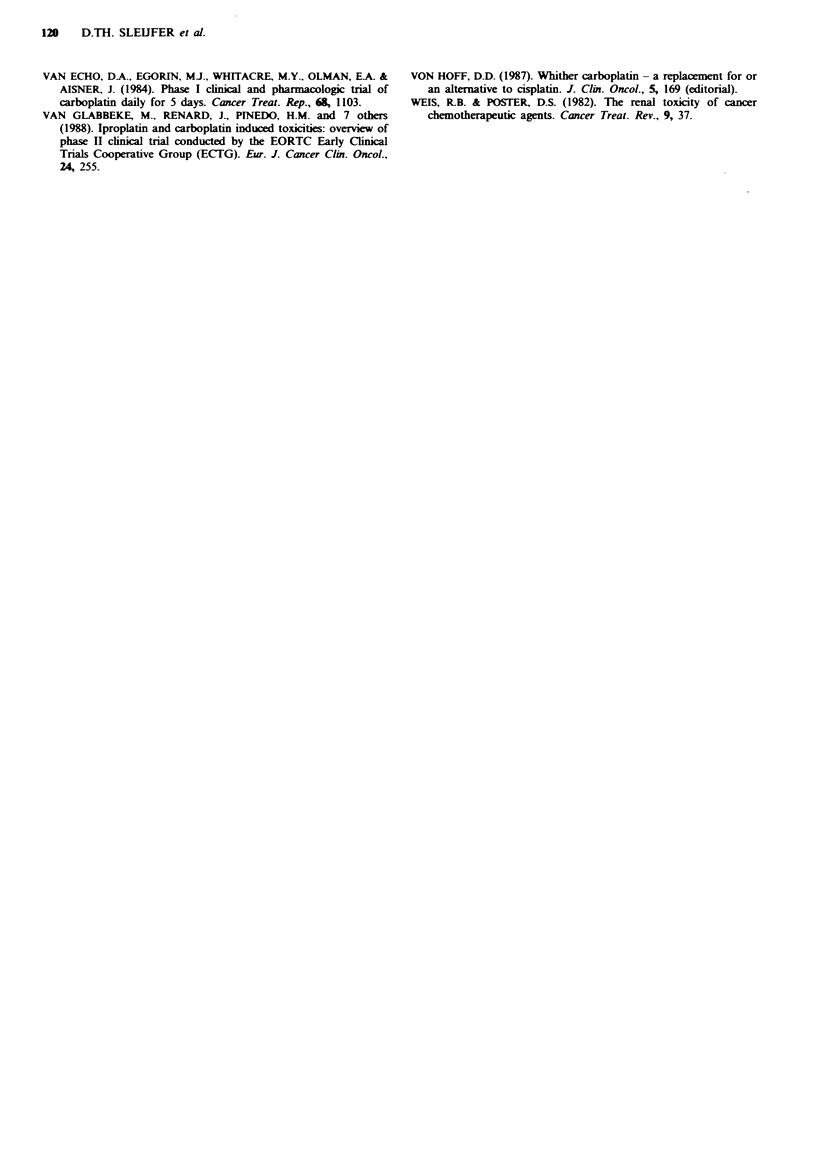# Acute and cumulative effects of carboplatin on renal function.

**DOI:** 10.1038/bjc.1989.233

**Published:** 1989-07

**Authors:** D. T. Sleijfer, E. F. Smit, S. Meijer, N. H. Mulder, P. E. Postmus

**Affiliations:** Department of Internal Medicine, University Hospital, Groningen, The Netherlands.

## Abstract

Carboplatin, a cisplatinum analogue, has no reported nephrotoxicity in phase I/II studies, assessed by creatinine clearance. We prospectively determined renal function in 10 untreated lung cancer patients with normal baseline renal function, treated with carboplatin 400 mg m-2 day 1 and vincristine 2 mg day 1 and 8 every 4 weeks (max. five cycles) by means of clearance studies with 125I-sodium thalamate and 131I-hippurate to determine GFR and ERPF respectively. Tubular damage was monitored by excretion of tubular enzymes and relative beta 2-microglobulin clearance. During the first course no changes in renal function were seen. After the second course a significant fall in GFR and ERPF started, ultimately leading to a median decrease in GFR of 19.0% (range 6.8-38.7%) and in ERPF of 14% (range 0-38.9%). No increases in the excretion of tubular enzymes or changes in the relative beta 2-microglobulin clearances were seen. We conclude from our data that carboplatin causes considerable loss of renal function. Monitoring renal function in patients treated with multiple courses of carboplatin is warranted.


Br. J. Cancer (1989), 60, 116–120 © The Macmillan Press Ltd., 1989

### Acute and cumulative effects of carboplatin on renal function

D.Th. Sleijfer, E.F. Smit, S. Meijer, N.H. Mulder & P.E. Postmus

*Department of Internal Medicine, Divisions of Medical Oncology, Nephrology and Pulmonology, University Hospital Groningen, Oostersingel 59, 9713 EZ Groningen, The Netherlands.*

**Summary** Carboplatin, a cisplatinum analogue, has no reported nephrotoxicity in phase I/II studies, assessed by creatinine clearance. We prospectively determined renal function in 10 untreated lung cancer patients with normal baseline renal function, treated with carboplatin 400 mg m$^{-2}$ day 1 and vincristine 2 mg day 1 and 8 every 4 weeks (max. five cycles) by means of clearance studies with $^{125}$I-sodium thalamate and $^{131}$I-hippurate to determine GFR and ERPF respectively. Tubular damage was monitored by excretion of tubular enzymes and relative $\beta_2$-microglobulin clearance. During the first course no changes in renal function were seen. After the second course a significant fall in GFR and ERPF started, ultimately leading to a median decrease in GFR of 19.0% (range 6.8–38.7%) and in ERPF of 14% (range 0–38.9%). No increases in the excretion of tubular enzymes or changes in the relative $\beta_2$-microglobulin clearances were seen. We conclude from our data that carboplatin causes considerable loss of renal function. Monitoring renal function in patients treated with multiple courses of carboplatin is warranted.

The introduction of cisplatin (CDDP) in the early seventies resulted in a major step forward in anticancer chemotherapy (Carter *et al.*, 1984). However, cisplatin has a narrow therapeutic index especially in regard to nephrotoxicity, limiting the clinical utility of this agent (Madias & Harrington, 1978). Several ways have been employed to overcome this problem. Although therapeutic index of CDDP has improved with the use of such manoeuvres, the drug does remain nephrotoxic (Al-Sarraf *et al.*, 1983; Ozols *et al.*, 1984; Markham *et al.*, 1985; Bodenner *et al.*, 1986; Elferink *et al.*, 1986; Offerman *et al.*, 1985). An alternative approach was the synthesis of analogues of cisplatin with the aim to find Pt-complexes with less nephrotoxicity and more or comparable antitumour activity (Burchenal *et al.*, 1979). About 2,000 second generation Pt-compounds have been synthesised and screened for cytotoxicity. Only a few have been selected for clinical evaluation, of which carboplatin (CBDCA, JM8) probably is the most promising. In human and animal studies carboplatin has demonstrated increased haematological toxicity compared to CDDP, but it is less emetogenic and has little or no oto- or neurotoxicity and no nephrotoxicity even in the absence of forced diuresis (Lelieveld *et al.*, 1984; Van Glabbeke *et al.*, 1988). In these studies the renal function was measured by monitoring serum creatinine and creatinine clearances. However, the determination of creatinine as a reflection of the glomerular filtration rate has proved to be a relatively insensitive method to monitor CDDP-induced renal damage (Meijer *et al.*, 1983; Daugaard *et al.*, 1988). Moreover, using $^{52}$Cr EDTA clearances, Calvert *et al.* (1982) were also unable to identify CBDCA as a nephrotoxic drug.

In this study we prospectively determined changes in glomerular filtration rate and effective renal plasma flow by the more sensitive method developed by Donker *et al.* (1977) in 10 patients treated with standard dose carboplatin. The possible tubular damage was monitored by measuring the excretion of tubular enzymes.

### Methods

### *Patients and therapy*

Ten patients, one female, nine male, were studied. All had histologically proved lung cancer (eight small cell lung cancer, one squamous cell carcinoma, one endobronchial carcinoid). One patient was pretreated with s.c. infusion of interferon, all others were previously untreated. Their age ranged from 48 to 69 years (mean 58). All had a normal serum creatinine level $<120\,\mu$mol l$^{-1}$, were normotensive, not salt restricted, and did not use other potentially nephrotoxic medication.

All patients were treated with carboplatin 400 mg m$^{-2}$ day 1 and vincristine 2 mg day 1 and 8 every 4 weeks. Carboplatin was dissolved in 250 ml of glucose 5% and given as a 30 min i.v. infusion on day 1. Vincristine was given as bolus injection. No pre- or post-hydration was given.

Seven responding patients received the maximum of five courses. The treatment had to be stopped in two patients after three and in one patient after two courses, because of tumour progression.

### *Renal function studies*

Glomerular filtration rate (GFR) and effective renal plasma flow (ERPF) were measured simultaneously in supine position with radioisotopes. ERPF was determined by measuring the clearance of $^{131}$I-hippuran ($I \times V/P$) and GFR by the clearance of $^{125}$I-iothalamate ($U \times V/P$) ($I$=counts per minute of 1 ml sustaining solution, $V$=infusion volume or urine volume in ml per minute, $P$=counts per minute in 2 ml of plasma and $U$=counts per minute in 2 ml urine). After a standard primary dose and sustaining infusion for 2 h, 1-h clearances were determined for acute effects and 2-h clearances for cumulative effects. For the latter, values are the mean of two 2-h clearances, which were corrected for standard body surface area. Errors in GFR introduced by incomplete collection of urine were corrected to a method previously described. The day to day variation of GFR is $\leqslant$2% and of ERPF $\leqslant$5% (Donker *et al.*, 1977). Filtration fraction (FF) was calculated as the quotient of GFR and ERPF.

Creatinine clearances were also corrected for incomplete collection of urine, using the same method as mentioned above.

These variables were studied before and during four hours after the first carboplatin infusion in order to determine acute effects on renal function. Cumulative effects were also measured during 4 h 4 weeks after each course, just before the administration of the following courses, i.e. on days 29, 57, 85, 113 and 141.

During renal function studies urine was collected hourly for the determination of creatinine, LDH, alkaline phosphatase (ALP), gamma-GT and $\beta_2$-microglobulin. Serum and urine creatinine, urine LDH, ALP and gamma-GT were determined with standard automatic techniques. $\beta_2$-Microglobulin concentration in plasma and urine was determined by a radioimmunosorbent technique according to Evrin *et al.* (1971). Next the ratio of respectively LDH, ALP, gamma-GT and creatinine (U per gram) were calculated. For $\beta_2$-

Correspondence: D.Th. Sleijfer.
Received 23 November 1988, and accepted in revised form 6 March 1989.

microglobulin the relative clearance in respect to creatinine clearance was calculated.

*Statistics*

Statistical analysis was performed with Wilcoxon's test for paired observations (two-sided); $P<0.05$ was considered indicative of a significant difference between groups.

### Results

*Acute effects*

The pretreatment values (A) of ERPF, GFR and FF of all patients are listed in Table I. Also depicted are the nadir values of ERPF and values of GFR and FF corresponding with the nadir values of ERPF after the first carboplatin infusion (B). Also, corresponding creatinine clearances are listed in Table I. There were no statistically significant changes in the GFR, either by the radiochemical method or creatinine clearances, and ERPF. For tubular enzymes no changes were seen. Relative clearance of $\beta_2$-microglobulin increased in the 4 h after carboplatin infusion, but remained within the normal range.

*Cumulative effects*

Absolute and relative changes in GFR and ERPF during the whole treatment are given in Table II and Figure 1, respectively. Corresponding creatinine clearances are also given. Four weeks after the first administration of carboplatin there is still no significant change in ERPF, GFR and FF, but deterioration of the renal function as measured by the radioisotope clearances occurs after the second course. Out of nine patients still on study 4 weeks after two courses, seven had a fall in GFR >2% ($P<0.02$), median −7.5%, range +1.9% to −36.1%. In three patients ERPF decreased >5% as opposed to pretreatment values; median −3.4%, range +24.1% to −40.0% ($P<0.05$). The ultimate decrease after five courses ($n=7$) ranges from 6.8% to 38.7% for GFR (median 19.0%) ($P<0.02$) and for ERPF 1.6% to 38.9% (median 14%) ($P<0.02$). These changes could not be explained by alteration in body weight of individual patients.

Although creatinine clearances showed a tendency to decrease after course 2, this change was not significant ($P>0.1$). Moreover, 4 weeks after the fourth course creatinine clearances retuned to baseline values, with the exception of those in patient number 10. After five courses no significant difference as opposed to pre-treatment values were found.

During the observation period no significant changes in serum creatinine were found. In regard to tubular enzymes and relative $\beta_2$ clearance we could not detect significant changes. Also, none of the patients developed proteinuria.

### Discussion

The most serious side effect of cisplatin is nephrotoxicity. After multiple courses of CDDP, a decrease of about 40% in creatinine clearance has been reported (Meijer *et al.*, 1983; Dentino *et al.*, 1978). The study of Meijer *et al.* (1983), using the same method as used in this report, showed a median decrease in GFR and ERPF of both 23% after induction chemotherapy containing CDDP for non-seminomatous testicular cancer. The cisplatin analogue carboplatin has no reported nephrotoxicity at conventional dose levels. The reduced protein binding (Van Echo *et al.*, 1984; Gaver *et al.*, 1987) and greater stability of carboplatin in body fluids and therefore increased renal excretion compared to cisplatin are supposed to account for the absence of nephrotoxicity (Harland *et al.*, 1984; Sharma *et al.*, 1983). Also, in animal models, carboplatin enhanced nuclear protein phosphorylation in tumour cells more than CDDP did, but caused much less protein phosphorylation in the normal liver and kidney cells. This suggests some selective toxicity towards tumour cells and may in part explain the decreased nephrotoxicity of carboplatin (Harrap *et al.*, 1980). Therefore, carboplatin has been recommended as an alternative to cisplatin in patients with impaired renal function or in those who cannot receive the hydration required for conventional cisplatin administration (Von Hoff, 1987).

There have been sporadic observations of renal function deterioration after multiple courses of carboplatin (Calvert *et al.*, 1982; Van Glabbeke *et al.*, 1988; Rozenczweig *et al.*, 1983; Leyvraz *et al.*, 1985). Also, the high dose ($\geq 800\,mg\,m^{-2}$) study of Gore *et al.* (1987) showed a fall in GFR of >25% in 55% of courses, as measured by ($^{51}Cr$) EDTA clearances.

Since vincristine has no reported nephrotoxicity (Schilsky, 1982; Weis & Poster, 1982), we conclude from the data of our study that carboplatin has a cumulative dose related nephrotoxic effect, as there was no decrease in renal function parameters after the first course, but an impressive fall in GFR and ERPF after the second course, ultimately leading to a decrease of 38% after five courses in some patients. The fall in GFR may be clinically important because the degree of myelosuppression probably depends on GFR (Egorin *et al.*, 1984; Fish *et al.*, 1987; Egorin *et al.*, 1985). In our patients, however, we did not find cumulative haematological toxicity despite this decrease in GFR. Even the patient with a 38% reduction in GFR did not have severe myelosuppression.

This study also shows the superiority of the radiochemical

**Table I** ERPF and GFR (creatinine clearance), all in $ml\,min^{-1}\,1.73\,m^{-2}$ before (A) and after (B) first carboplatin infusion

| | *ERPF* | | *GFR (creatinine clearance)* | | *FF* | |
|---|---|---|---|---|---|---|
| *Patient* | *A* | *B* | *A* | *B* | *A* | *B* |
| 1 | 660 | 601 | 121 (106) | 119 (96) | 0.18 | 0.20 |
| 2 | 375 | 395 | 118 (107) | 136 (95) | 0.30 | 0.35 |
| 3 | 401 | 397 | 155 (115) | 158 (131) | 0.39 | 0.39 |
| 4 | 293 | 309 | 87 (80) | 92 (75) | 0.28 | 0.30 |
| 5 | 312 | 322 | 83 (98) | 94 (82) | 0.27 | 0.29 |
| 6 | 271 | 235 | 80 (82) | 81 (72) | 0.30 | 0.34 |
| 7 | 319 | 315 | 105 (116) | 102 (107) | 0.33 | 0.32 |
| 8 | 599 | 517 | 123 (118) | 130 (116) | 0.21 | 0.23 |
| 9 | 301 | 309 | 102 (110) | 113 (122) | 0.34 | 0.37 |
| 10 | 558 | 526 | 143 (134) | 154 (114) | 0.26 | 0.29 |
| Median | 388 | 358.5 | 111.5 (108.5) | 121.5 (101.5) | 0.29 | 0.31 |
| Mean | 408.9 | 398 | 111.7 (106.6) | 117.9 (101) | 0.286 | 0.308 |
| s.d. | 143.06 | 125.88 | 25.14 (16.5) | 26.39 (20.3) | 0.0615 | 0.0596 |
| s.e.m. | 45.24 | 39.81 | 7.95 (5.21) | 8.34 (6.42) | 0.0194 | 0.0188 |

**Table II** Absolute changes in GFR (creatinine clearance) and ERPF, all in ml min$^{-1}$ 1.73 m$^{-2}$ after multiple courses

| Patient | Course number 0 | 1 | 2 | 3 | 4 | 5 |
|---|---|---|---|---|---|---|
| *GFR (creatinine clearance)* | | | | | | |
| 1 | 121 (106) | 124 (112) | 95 (79) | 98 (93) | 101 (105) | 96 (129) |
| 2 | 118 (107) | 137 (124) | 113 (101) | | | |
| 3 | 155 (115) | 116 (100) | 99 (116) | 97 (109) | 94 (179) | 95 (68) |
| 4 | 87 (80) | 97 (125) | 82 (93) | 91 (97) | 87 (111) | 81 (59) |
| 5 | 83 (98) | 90 (114) | 78 (101) | | | |
| 6 | 80 (82) | 79 (85) | 74 (87) | 73 (84) | 72 (81) | 62 (91) |
| 7 | 105 (116) | 102 (113) | 107 (72) | 101 (109) | 89 (107) | 85 (91) |
| 8 | 123 (118) | 131 (102) | 124 (143) | 120 (145) | 119 (123) | 114 (127) |
| 9 | 102 (110) | 116 (120) | | | | |
| 10 | 143 (134) | 111 (90) | 110 (90) | 98 (79) | 103 (50) | 122 (116) |
| Median | 111.5 (110) | 116 (112.5) | 99 (93) | 98 (97) | 94 (111) | 95 (91) |
| Mean | 111.7 (106.5) | 110.3 (108.5) | 98 (99) | 96.9 (102.3) | 95.0 (108) | 93.6 (97) |
| s.d. | 25.14 (16.5) | 18.35 (13.8) | 17.20 (22.5) | 13.90 (22.0) | 14.75 (39.5) | 10.26 (27.8) |
| s.e.m. | 7.95 (5.21) | 5.80 (4.37) | 5.73 (7.49) | 5.25 (8.31) | 5.58 (14.94) | 7.66 (10.5) |
| *ERPF* | | | | | | |
| 1 | 660 | 540 | 396 | 413 | 423 | 403 |
| 2 | 375 | 492 | 424 | | | |
| 3 | 401 | 470 | 407 | 360 | 320 | 310 |
| 4 | 293 | 331 | 283 | 350 | 288 | 290 |
| 5 | 312 | 376 | 302 | | | |
| 6 | 271 | 260 | 260 | 254 | 249 | 234 |
| 7 | 319 | 354 | 396 | 372 | 330 | 314 |
| 8 | 599 | 554 | 499 | 531 | 558 | 555 |
| 9 | 301 | 452 | | | | |
| 10 | 558 | 385 | 374 | 348 | 382 | 398 |
| Median | 388 | 418.5 | 396 | 360 | 330 | 314 |
| Mean | 408.9 | 421.4 | 371.2 | 375.4 | 364.3 | 357.7 |
| s.d. | 143.06 | 95.50 | 76.30 | 83.67 | 102.90 | 105.41 |
| s.e.m. | 45.24 | 30.20 | 25.43 | 31.63 | 38.89 | 39.84 |

**Figure 1** Mean percent changes in GFR (ml min$^{-1}$ 1.73 m$^{-2}$) (**a**) and ERPF (ml min$^{-1}$ 1.73 m$^{-2}$) (**b**) after multiple courses of carboplatin (mean ± s.e.).

method to determine GFR as opposed to creatinine clearance in order to monitor Pt induced renal damage. The latter method failed to detect a significant change in GFR after multiple courses of carboplatin. A possible explanation for this finding is provided by Meyer *et al.* (1983). A significant fall in GFR (median 23%) was seen after combination chemotherapy containing CDDP, without a rise in serum creatinine. They suggested that the chemotherapy interfered with enzymic systems required for creatinine production.

The mode of action of Pt-induced nephrotoxicity remains unknown. Offerman *et al.* (1984) have shown that the acute effect of CDDP on renal function is a fall in ERPF preceding a similar change in GFR. Also, in experimental models of renal failure following intoxication with heavy metals, in the initial phase a reduction of renal blood flow can be found. In our study no such phenomenon could be found, as during the first course neither a fall in GFR nor in ERPF was seen. Since a simultaneous decrease in both GFR and ERPF occurred 4 weeks after course 2, we can only speculate, but not exclude, whether such a sequence of events did take place. Also, the intracellular presence of reactive Pt-compounds in the kidney is suggested to relate to this toxicity (Harland *et al.*, 1984; Stewart *et al.*, 1985). Therefore tubular damage might play an important role in Pt-induced nephrotoxicity. The reported renal uptake of carboplatin does not differ substantially from that for CDDP (Lelieveld *et al.*, 1984; Owens *et al.*, 1985). CDDP induces tubular damage in most patients but we could not find signs of tubular damage after carboplatin administration as measured by the urinary excretion of tubular enzymes, because of infrequent sampling. For the evaluation of tubular damage timing of specimen collection plays an important role (Goren *et al.*, 1987). As reported for cisplatin (Goren *et al.*, 1986) and carboplatin (Egorin *et al.*, 1984), urinary enzymes can peak as late as several days after the administration of the drug. Shillen *et al.* (1988) collected weekly specimens for the evaluation of excretion of urinary protein and enzymes in patients receiving multiple courses of carboplatin 400 mg m$^{-2}$. In some of their patients urinary enzymes peaked 2 weeks after the administration of the drug. Therefore, our results might be misleading in that they were obtained for a period of only 4 h after administration.

We conclude from our data that carboplatin exerts dose-related cumulative renal damage. Careful monitoring, especially with regard to myelosuppression, in patients with impaired renal function or those pretreated with Pt containing regimens is therefore warranted. The observed reduction in renal function after carboplatin is in the same range compared to patients treated with cisplatin with saline diuresis (Meijer *et al.*, 1983). Although it has not been excluded that hyperhydration during carboplatin treatment could prevent the nephrotoxic effects, the value of administering this cytostatic drug on an outpatient base would disappear.

This study was supported by grant C84-503 of the Dutch Kidney Foundation (Nierstichting Nederland). The authors thank Willy Bruins-van der Weij for secretarial support and Aly Drent-Bremer for technical assistance.

### References

AL-SARRAF, M., FLETCHER, W., OISHI, H. and 5 others (1983). Cisplatin hydration with and without mannitol diuresis in refractory disseminated malignant melanoma: a southwest oncology group study. *Cancer Treat. Rep.*, **66**, 31.

BODENNER, D.L., DEDON, P.C., KENG, P.C., KATZ, J.C. & BORCH, R.F. (1986). Selective protection against cis-diamminechloride-platinum (II)-induced toxicity in kidney, gut and bone marrow by diethyldithiocarbamate. *Cancer Res.*, **46**, 2751.

BURCHENAL, J.H., KALAHER, K., DEW, K. & LOKYS, L. (1979). Rationale for development of platinum analogs. *Cancer Treat. Rep.*, **65**, 1493.

CALVERT, A.H., HARLAND, S.J., NEWELL, D.R. and 9 others (1982). Early clinical studies with *cis*-diammine-1,1-cyclobutane dicarboxylate platinum II. *Cancer Chemother. Pharmacol.*, **9**, 140.

CARTER, S.K. (1984). Cisplatin – past, present and future. In *Platinum Coordination Complexes in Cancer Chemotherapy*, Hacker, M.P., Douple, E.B. & Krakoff, I.H. (eds) p. 359. Martinus Nijhoff: Boston.

DAUGAARD, G., ROSSING, N. & RORTH, M. (1988). Effects of cisplatin on different measures of glomerular function in the human kidney with special emphasis on high dose. *Cancer Chemother. Pharmacol.*, **21**, 163.

DENTINO, M., LUFT, F.C., YUM, M.N., WILLIAMS, S.D. & EINHORN, L.H. (1978). Long term effect of *cis*-diamminochloride platinum (CDDP) on renal function and structure in man. *Cancer*, **41**, 1274.

DONKER, A.J.M., VAN DER HEM, G.K., SLUITER, W.J. & BEEKHUIS, A. (1977). A radioisotope method for simultaneous determination of the glomerular filtration rate and the effective renal plasma flow. *Neth. J. Med.*, **20**, 97.

EGORIN, M.J., VAN ECHO, D.A., TIPPING, S.J. and 4 others (1984). Pharmacokinetics and dosage reduction of *cis*-diammine(1,1-cyclobutanedicarboxylato) platinum in patients with impaired renal function. *Cancer Res.*, **44**, 5432.

EGORIN, M.J., VAN ECHO, D.A., OLMAN, E.A., WHITACRE, M.Y., FORREST, A. & AISNER, J. (1985). Prospective validation of a pharmacologically based dosing scheme for the *cis*-diammine dichloroplatinum (II) analogue cis(diammine (1,1) cyclobutanedicarboxylatoplatinum. *Cancer Res.*, **45**, 6502.

ELFERINK, F., VAN DER VIJGH, W.J.F. KLEIN, I. & PINEDO, H.M. (1986). Interaction of cisplatin and carboplatin with sodium thiosulfate: reaction rates and protein binding. *Clin. Chem.*, **32**, 641.

EVRIN, P.E., PETERSON, P.A., WIDE, L. & BERGGARD, I. (1971). Radioimmunoassay of $\beta_2$ microglobulin in human biological fluids. *Scand. J. Clin. Lab. Invest.*, **28**, 439.

FISH, R.G., SHELLEY, M.D., GRIFFITH, H. & ADAMS, M. (1987). Letter. *Cancer Res.*, **47**, 3606.

GAVER, R.C., GEORGE, A.M. & DAB, G. (1987). *In vitro* stability, plasma protein binding and blood cell partitioning of $^{14}C$ carboplatin. *Cancer Chemother. Pharmacol.*, **20**, 271.

GORE, M.E., CALVERT, A.H. & SMITH, I.E. (1987). High dose carboplatin in the treatment of lung cancer and mesothelioma: a phase I dose escalation study. *Eur. J. Cancer Clin. Oncol.*, **23**, 1391.

GOREN, M.P., WRIGHT, R.K. & HOROWITZ, M.E. (1986). Cumulative renal tubular damage associated with cisplatin nephrotoxicity. *Cancer Chemother. Pharmacol.*, **18**, 69.

GOREN, M.P., FORASTIERE, A.A., WRIGHT, R.K. and 5 others (1987). Carboplatin (CBDCA), iproplatin (CHIP) and high dose cisplatin in hypertonic saline evaluated for tubular nephrotoxicity. *Cancer Chemother. Pharmacol.*, **19**, 57.

HARLAND, S.J., NEWELL, D.R., SIDDIK, Z.H., CHADWICK, R., CALVERT, A.H. & HARRAP, K.R. (1984). Pharmacokinetics of cis-diammine-1,1-cyclobutane dicarboxylate platinum (II) in patients with normal and impaired renal function. *Cancer Res.*, **44**, 1693.

HARRAP, K.R., JONES, M., WILKINSON, C.R. and 5 others (1980). Antitumor, toxic and biochemical properties of cisplatin and eight other platinum complexes. In *Cisplatin Current Status and New Developments*, Prestayko, A.W., Crooke, S.T. & Carter, S.K. (eds). Academic Press: London.

LELIEVELD, P., VAN DER VIJGH, W.J.F., VELDHUIZEN, R.W. and 4 others (1984). Preclinical studies on toxicity, antitumour activity and pharmacokinetics of cisplatin and three recently developed derivates. *Eur. J. Cancer Clin. Oncol.*, **20**, 1087.

LEYVRAZ, S., OHNUMA, T., LASSUS, M. & HOLLAND, J.F. (1985). Phase I study of carboplatin in patients with advanced cancer, intermittent intravenous bolus and 24-hour infusion. *J. Clin. Oncol.*, **3**, 1385.

MADIAS, N.E. & HARRINGTON, J.T. (1978). Platinum nephrotoxicity. *Am. J. Med.*, **65**, 307.

MARKMAN, M., CLEARY, S. & HOWELL, S.B. (1985). Nephrotoxicity of high-dose intracavitary cisplatin with intravenous thiosulfate protection. *Eur. J. Cancer Clin. Oncol.*, **21**, 1015.

MEIJER, S., SLEIJFER, D.TH., MULDER, N.H. and 7 others (1983). Some effects of combination chemotherapy with cis-platinum on renal function in patients with nonseminomatous testicular carcinoma. *Cancer*, **51**, 2035.

OFFERMAN, J.J.G., MEYER, S., SLEIJFER, D.TH. and 5 others (1984). Acute effects of cisdiamminedichloroplatinum (CDDP) on renal function. *Cancer Chemother. Pharmacol.*, **12**, 36.

OFFERMAN, J.J.G., MULDER, N.H., SLEIJFER, D.TH. and 4 others (1985). Influence of captopril on cis-diammine-dichloro-platinum induced renal toxicity. *Am. J. Nephrol.*, **5**, 433.

OWENS, S.E., THATCHER, N. SHARMA, H. and 8 others (1985). *In vitro* distribution studies of radioactively labelled platinum complexes: *cis*-dichlorodiammine platinum (II), *cis-trans*-dichlorodihydroxy-*bis*-(isopropylamine) platinum (IV), *cis*-dichloro-*bis*-cyclo-propylamine platinum (II) and *cis*-diammine 1,1-cyclobutanedicarboxylate platinum (II) in patients with malignant disease, using a gamma camera. *Cancer Chemother. Pharmacol.*, **14**, 253.

OZOLS, R.F., CORDON, B.J., JACOB, J., WESLEY, M.M., OSTCHEGA, Y. & YOUNG, R.C. (1984). High-dose cisplatin in hypertonic saline. *Ann. Intern. Med.*, **100**, 19.

ROZENCZWEIG, M., NICAISE, C. & BEIR, M. (1983). Phase I study of carboplatin given on a five-day intravenous schedule. *J. Clin. Oncol.*, **1**, 621.

SHILLEN, A.W., BUAMAH, P.K., CANTWELL, B.M.J., CORNELL, C., HODSON, A.W. & HARRIS, A.L. (1988). Urinary protein and enzyme excretion in patients receiving chemotherapy with the cisplatin analogs carboplatin (CBDCA, JM8) and iproplatin (CHIP, JM9). *Cancer Chemother. Pharmacol.*, **22**, 228.

SCHILSKY, R.L. (1982). Renal and metabolic toxicities of cancer chemotherapy. *Semin. Oncol.*, **9**, 75.

SHARMA, H., THATCHER, N., BEAR, J. and 6 others (1983). Blood clearance of radioactively labelled *cis*-diammine 1,1-cyclobutane dicarboxylate platinum (II) (CBDCA) in cancer patients. *Cancer Chemother. Pharmacol.*, **11**, 5.

STEWART, D.J., MIKHAEL, N.Z., NANJI, A.A. and 5 others (1985). Renal and hepatic concentrations of platinum: relationship to cisplatin time, dose and nephrotoxicity. *J. Clin. Oncol.*, **3**, 1251.

VAN ECHO, D.A., EGORIN, M.J., WHITACRE, M.Y., OLMAN, E.A. & AISNER, J. (1984). Phase I clinical and pharmacologic trial of carboplatin daily for 5 days. *Cancer Treat. Rep.*, **68**, 1103.

VAN GLABBEKE, M., RENARD, J., PINEDO, H.M. and 7 others (1988). Iproplatin and carboplatin induced toxicities: overview of phase II clinical trial conducted by the EORTC Early Clinical Trials Cooperative Group (ECTG). *Eur. J. Cancer Clin. Oncol.*, **24**, 255.

VON HOFF, D.D. (1987). Whither carboplatin – a replacement for or an alternative to cisplatin. *J. Clin. Oncol.*, **5**, 169 (editorial).

WEIS, R.B. & POSTER, D.S. (1982). The renal toxicity of cancer chemotherapeutic agents. *Cancer Treat. Rev.*, **9**, 37.